# EGFR mutations and AKT phosphorylation are markers for sensitivity to combined MCL-1 and BCL-2/xL inhibition in non-small cell lung cancer

**DOI:** 10.1371/journal.pone.0217657

**Published:** 2019-05-31

**Authors:** Shawn J. Rice, Xin Liu, Hong-Gang Wang, Chandra P. Belani

**Affiliations:** 1 Penn State Hershey Cancer Institute, Hershey, Pennsylvania, United States of America; 2 Department of Pediatrics, Penn State College of Medicine, Hershey, Pennsylvania, United States of America; 3 Department of Medicine, Penn State College of Medicine, Hershey, Pennsylvania, United States of America; Roswell Park Cancer Institute, UNITED STATES

## Abstract

Lung cancer is among the common and deadly cancers. Although the treatment options for late-stage cancer patients have continued to increase in numbers, the overall survival rates for these patients have not shown significant improvement. This highlights the need for new targets and drugs to more effectively treat lung cancer patients. In this study, we characterize the MCL-1 inhibitor maritoclax alone or in combination with a BCL-2/xL inhibitor in a panel of lung cancer cell lines. BCL-2 family proteins, phosphorylated proteins, and apoptosis were monitored following the treatments. We found that maritoclax was effective at inhibiting growth in these lung cancer cells. We also establish that cell lines with EGFR mutations were most sensitive to the combined inhibition of MCL-1 and BCL-2/xL. In addition, a high level of phosphorylated AKT (S473) was identified as a marker for sensitivity to the combination treatment. This work has defined EGFR mutations and AKT phosphorylation as markers for sensitivity to combined MCL-1 and BCL-2/xL targeted therapy and establishes a rationale to explore multiple BCL-2 family members in patients who are refractory to EGFR inhibitor treatment. Our data support the design of a clinical trial that aims to employ inhibitors of the BCL-2 family of proteins in lung cancer patients.

## Introduction

Lung cancer accounts for over one-quarter of cancer-related mortalities and significant healthcare cost annually [1, 2]. The survival rate in lung cancer continues to be modest with little improvement over the past few decades [3, 4]. Additionally, the overall 5-year survival rate for lung cancer is 17%, however, when diagnosed early, stage I, that rate increased to 83% [5]. Current strategies for the prevention and treatment of lung cancer remain disappointing. Therapeutic options in lung cancer are numerous and continually expanding, however, their efficacy in late-stage patients is varied and often transient.

Anti-apoptotic BCL-2 family proteins (BCL-2, BCL-xL, and MCL-1) are emerging as important factors for drug resistance in lung cancer and may represent new targets for treatment. These proteins function to prevent apoptosis through the inhibition of the mitochondrial outer-membrane permeabilization (MOMP), which is determined by the balance between anti- and pro-apoptotic BCL-2 family proteins that interact with each other through shared BCL-2 homology (BH) domains [6]. A low ratio of anti- to pro-apoptotic BCL-2 family members primes cells for apoptosis, and predicts sensitivity to chemotherapy drugs [7–9]. Conversely, excessive protein levels of anti-apoptotic BCL-2 proteins potentiate a drug resistance phenotype. In lung cancer, cells which have high levels of the pro-apoptotic member BIM (protein and mRNA expression) or those with a low ratio of anti- to pro-apoptotic members following EGFR inhibitor treatment, were more sensitive to the agent [10, 11]. High BIM levels were also associated with enhanced overall response rate (ORR) and progression-free survival (PFS) relative to patients with low or moderate BIM in NSCLC patients treated with the EGFR inhibitor erlotinib [12].

These *in vitro* and clinical data suggest that targeting anti-apoptotic BCL-2 proteins could improve the efficacy of drugs already used in the clinic. A BCL-2/BCL-xL-specific inhibitor navitoclax (ABT-263, parent compound ABT-737) has been developed and tested in clinical trials. This drug has shown *in vitro* and *in vivo* efficacy in combination with targeted therapies like EGFR inhibitors in EGFR mutation-positive NSCLC or BRAF/MEK inhibitors in BRAF mutation-positive melanomas [13–17]. Resistance to BCL-2 targeting, by small molecule inhibition or siRNA knockdown, often involves the activation of MCL-1 expression [18–20]. This highlights the importance of all the anti-apoptotic BCL-2 family proteins in drug resistance.

Marinopyrrole A (maritoclax) has recently been identified as a naturally occurring compound with the ability to inhibit the BIM-MCL-1 interaction, induce proteolytic degradation of MCL-1, and potentiate apoptosis of leukemia cells [21]. Subsequently, maritoclax has been shown to produce a similar effect in melanoma cells, which was enhanced when combined with the BCL-2/xL inhibitor, navitoclax [22]. Additional work has suggested that maritoclax may have efficacy in the many types of malignancies including lung cancer [23–25]. In this work, we characterize maritoclax in a panel of lung cancer cell lines with varied driver mutation backgrounds. We also characterize the combination of maritoclax and a BCL-2/xL inhibitor in the panel of cell lines. We identify EGFR-mutated cell lines as being most sensitive to the drug combination, and AKT phosphorylation as the marker for sensitivity to the combined inhibition of MCL-1 and BCL-2/xL.

## Materials and methods

### Cell culture

NCI-A549, NCI-H23, NCI-358, NCI-441, NCI-460, NCI-H1299, NCI-1650, NCI-H1755, and NCI-1975 cells were acquired from ATCC and propagated in RPMI 1650 media supplemented with 10% FBS and penicillin and streptomycin.

### Growth assay

Cells were plated on 96 well plates, twenty-four hours later cells were treated with the serially diluted drug as indicated in the figures. Forty-eight hours after treatment cells were assayed for growth using MTS reagent (Promega) according to the manufacturers’ specifications. Absorbance values were normalized to the DMSO treated wells and used to calculate IC_50_ values with GraphPad Prizm software.

### Western blots

Protein samples were isolated in RIPA buffer (Sigma) supplemented with protease and phosphates inhibitors (Sigma) prior to quantification with a BCA kit (Thermo Scientific). Total protein samples (15–20 μg) were separated through SDS-PAGE, electro-blotted to PVDF, blocked with 5% non-fat dry milk, incubated with primary antibodies and secondary antibodies (all antibodies from Cell Signaling Technologies), and detected with enhanced-chemiluminescent (ECL) substrate (Thermo Scientific). Bands were quantified by densitometry using ImageJ software (https://imagej.nih.gov/ij/).

### Apoptosis by flow cytometry

Cells were assayed for apoptosis by flow cytometry using AnnexinV-FITC and 7-Amino-Actinomycin D (7-AAD). Cells were treated as indicated for twenty-four hours, treated with trypsin, and incubated for at least 15 min in 1x Binding buffer (BD Biosciences) with Annexin V-FITC (BD Biosciences) and 7-AAD (BD Biosciences) according to manufacturers’ specifications. Samples were evaluated on BD Calibur flow cytometer (BD Biosciences) and the data were analyzed using WinMDI 2.8 software (http://www.cyto.purdue.edu/flowcyt/software/Winmdi.htm).

### Caspase 3/7 assay

Cells (10x10^3^) were seeded onto 96 well white-walled plates. After 24 hours, cells were treated in triplicate as indicated. Twenty-four hours later, an equal volume of Caspase 3/7Glo assay mix (Promega) was added to each well. Plates were mixed by shaking and assayed for luminescence using a Synergy HT plate reader (BioTek).

## Results

### Maritoclax-induced growth inhibition in NSCLC cell lines

**Fig 1:**
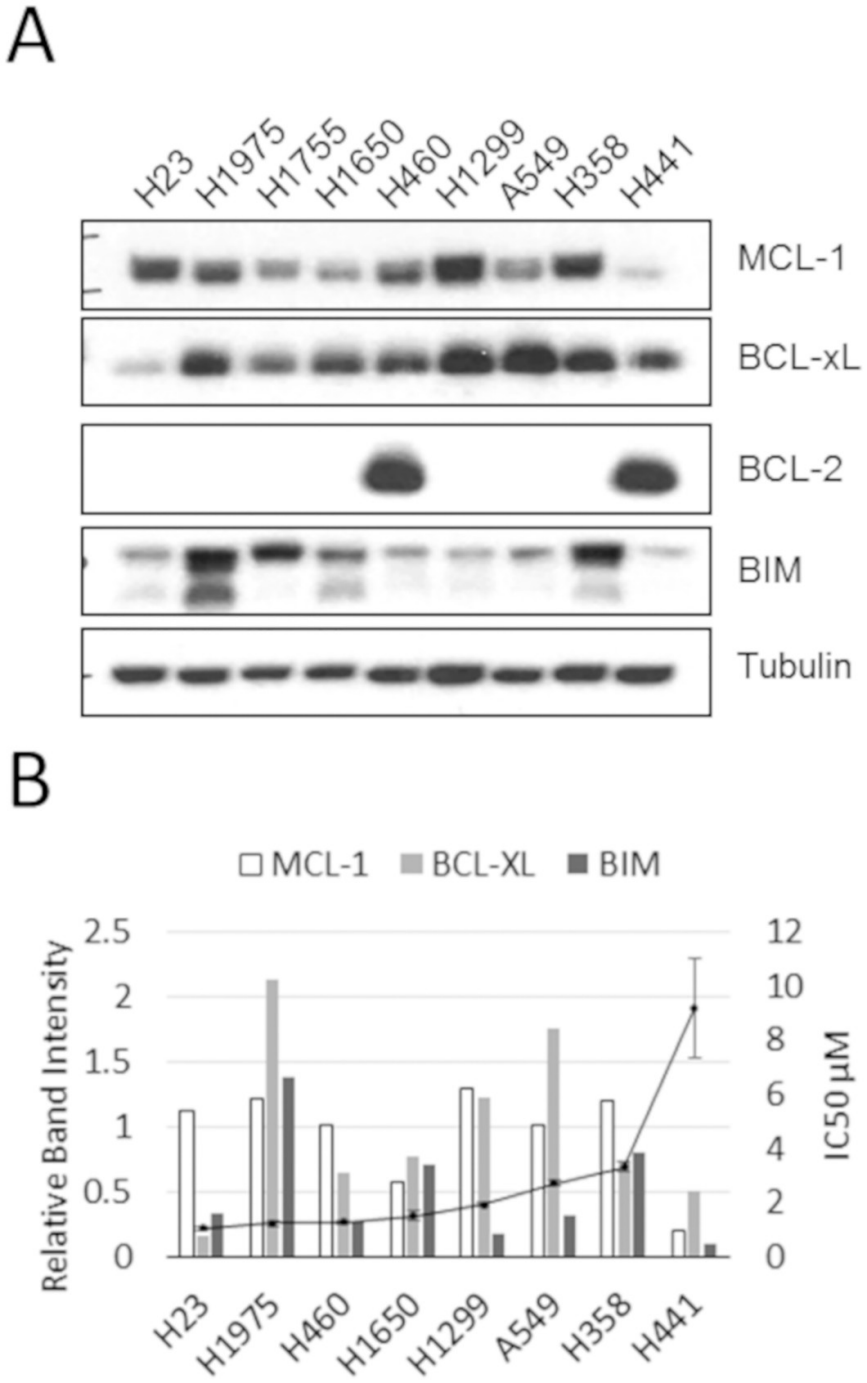
Efficacy of maritoclax for inhibiting growth in a panel of NSCLC cell lines. (A) Expression of BCL-2 family proteins in the various NSCLC cell lines was determined by western blot. Indicated cell lines were treated with varying concentrations of maritoclax for 48 hours, then assayed for viability with an MTS assay. (B) Indicated cell lines were treated with varying concentrations of maritoclax for 48 hours, then assayed for viability with an MTS assay. The 50% inhibition concentration for maritoclax was calculated from viability data (n = 3). The IC_50_ data is represented as a line graph on the secondary (right) axis and overlayed on the relative BCL-2 family protein level data from the western blot on the primary (left) axis.

A panel of nine NSCLC cell lines was selected for this study. The panel included cell lines with varied oncogenic mutations (Table 1) including BRAF (H1755), EGFR (H1650 and H1975), KRAS (A549, H23, H358, H441, and H460), and NRAS (H1299). The cell lines were evaluated by western blot for BCL-2, BCL-xL, MCL-1, and BIM protein expression. Similar to previous reports (18), MCL-1 and BCL-xL were detected in all cell lines, but the extremely low and high expression was seen in the H441 and H23 cells, respectively (Fig 1A). BIM was observed in all cell lines tested, while BCL-2 was detected in only H460 and H441 cells.

**Table 1:** Driver mutations in the NSCLC cell lines used in this study.

Cell line	Gene	AA Change
**A549**	KRAS	p.G12S
**H23**	KRAS	p.G12C
**H358**	KRAS	p.G12V
**H441**	KRAS	p.G12V
**H460**	KRAS	p.Q61H
**H1299**	NRAS	p.Q61K
**H1650**	EGFR	p.E749-A750del
**H1755**	BRAF	p.G469A
**H1975**	EGFR	p.T790M,p.L858R

Next, we tested the effect of the MCL-1 inhibitor, maritoclax, on our panel of NSCLC cell lines. Cell lines were treated individually with five concentrations of maritoclax and viability was assessed after 48 hours. Maritoclax treatment resulted in IC_50_ values between 1.1–9.2 μM (H23 and H441 cells, respectively) in the cell lines tested (Fig 1B). Maritoclax-associated growth inhibition was independent of driver gene mutations for the cell lines evaluated in this study. In addition, a complex relationship between maritoclax sensitivity and BCL-2 family proteins was observed in the cell lines (Fig 1B).

### Maritoclax enhances MCL-1 degradation in H23 cells

**Fig 2:**
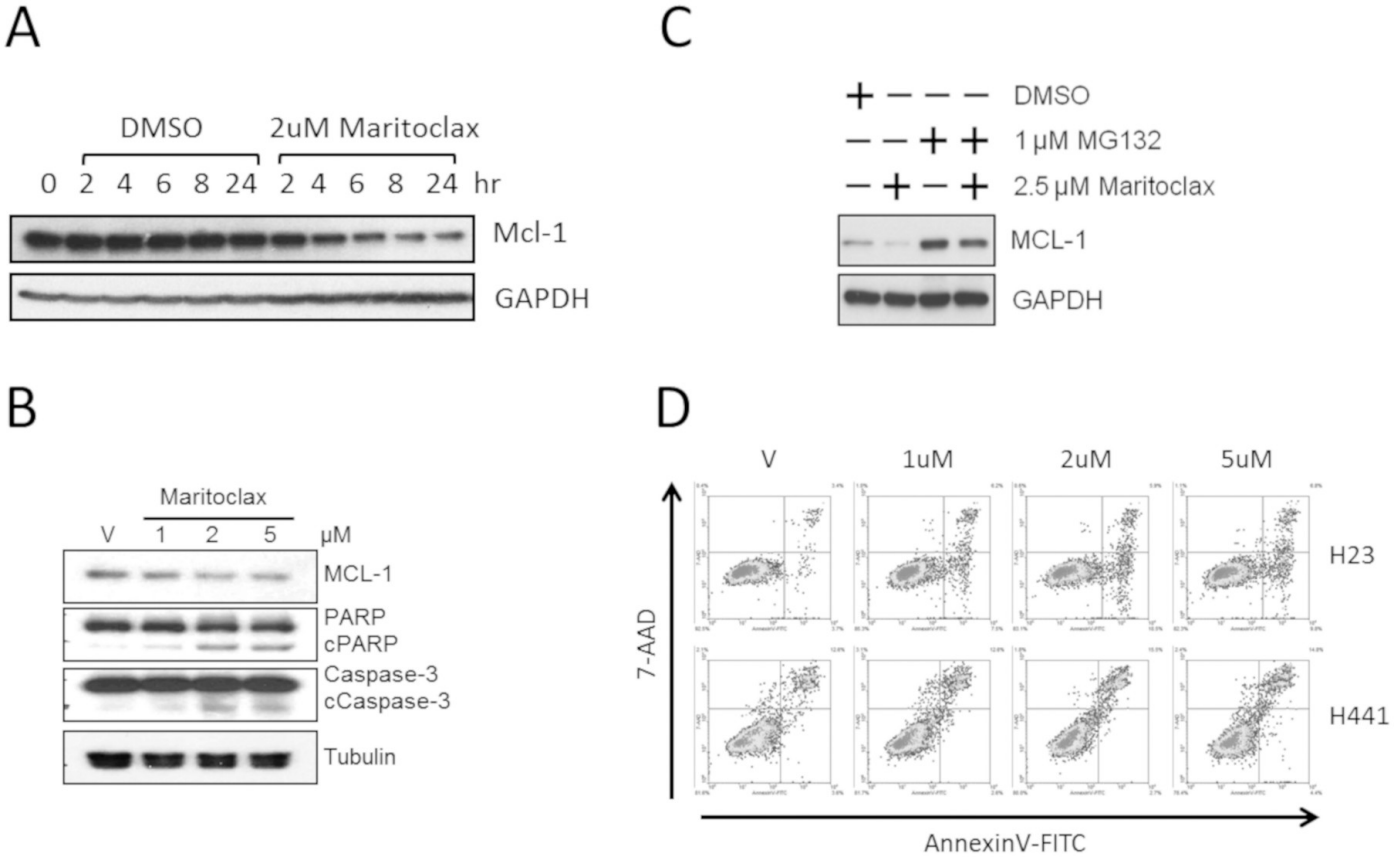
Maritoclax inhibits MCL-1 expression and induces apoptosis in H23 NSCLC cell line. (A) H23 cells were treated with 2 μM maritoclax for various times (0–24 hours). MCL-1 expression was evaluated by western blot. (B) H23 cells were treated with the indicated doses of maritoclax or DMSO only (V). After 24 hours, proteins were harvested and MCL-1 expression and the apoptosis markers cleaved PARP (cPARP) and cleaved Caspase-3 (cCaspase-3) were assessed by western blot. (C) H23 cells were treated with maritoclax alone or in combination with the proteasome inhibitor MG132 as indicated for 24 hours, prior to the determination of MCL-1 levels by western blot. (D) Induction of apoptosis by maritoclax in H23 was measured using an annexin-V/7-AAD assay quantified by flow cytometry. H23 cells were treated with indicated doses of maritoclax for 24 hours prior to incubation with annexin-V-FITC and 7-AAD and quantification by flow cytometry on a Calibur system (BD Biosciences). (n = 3).

We selected H23 cells to investigate the mechanism of action of maritoclax in lung cells because they were the most sensitive to the drug and had little endogenous BCL-xL and BCL-2 protein, which can compensate for MCL-1 loss [21]. H23 cells treated with maritoclax exhibited a dose and time-dependent decrease in MCL-1 protein levels (Fig 2A and 2B). Additionally, Inhibition of MCL-1 expression was also observed in H1975, H1650, and H1299 cells following maritoclax treatment (S1 Fig). In addition, the loss of MCL-1 was also associated with an increase in the apoptosis markers cleaved PARP and cleaved Caspase-3 (Fig 2B). Pharmacological inhibition of the proteasome complex in H23 cells by MG132 resulted in the accumulation of MCL-1 protein, which persisted after co-treatment with maritoclax (Fig 2C). Therefore, maritoclax-induced MCL-1 loss is dependent on the proteasome, which is in line with previous reports [21, 22].

Next, we asked if maritoclax induced apoptosis in NSCLC cells. First, we subjected H23 cells to maritoclax then assay cells for apoptosis after 24 hours using flow cytometry. Maritoclax potentiated a dose-dependent increase in annexin-V positive (apoptotic) cells while producing a muted effect in the resistant H441 cell line (Fig 2D). Maritoclax also enhanced caspase 3/7 activity (~2-fold) in H23 cells 24 hours after treatment, which match the annexin-V results (S2 Fig).

### Synergistic effect of combining maritoclax with a BCL-2/xL inhibitor

**Fig 3:**
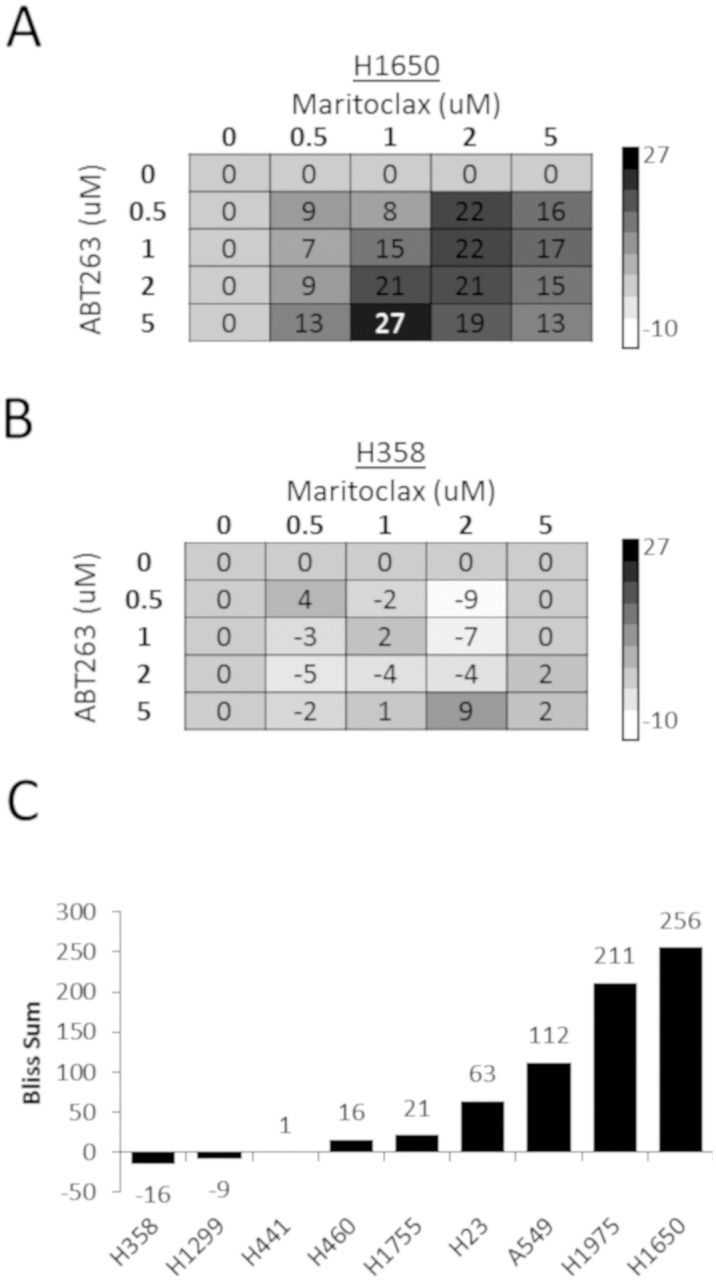
Maritoclax and ABT-263 synergy in the panel of NSCLC cell lines. (A-B) Synergy was evaluated using the Bliss Sum method (Materials and methods). (A) the Bliss matrixes for the least and most synergistic cell lines, H1650 (A) and H358 (B), respectively. (C) Comparison of Bliss Sum values for each cell line screened.

**Fig 4:**
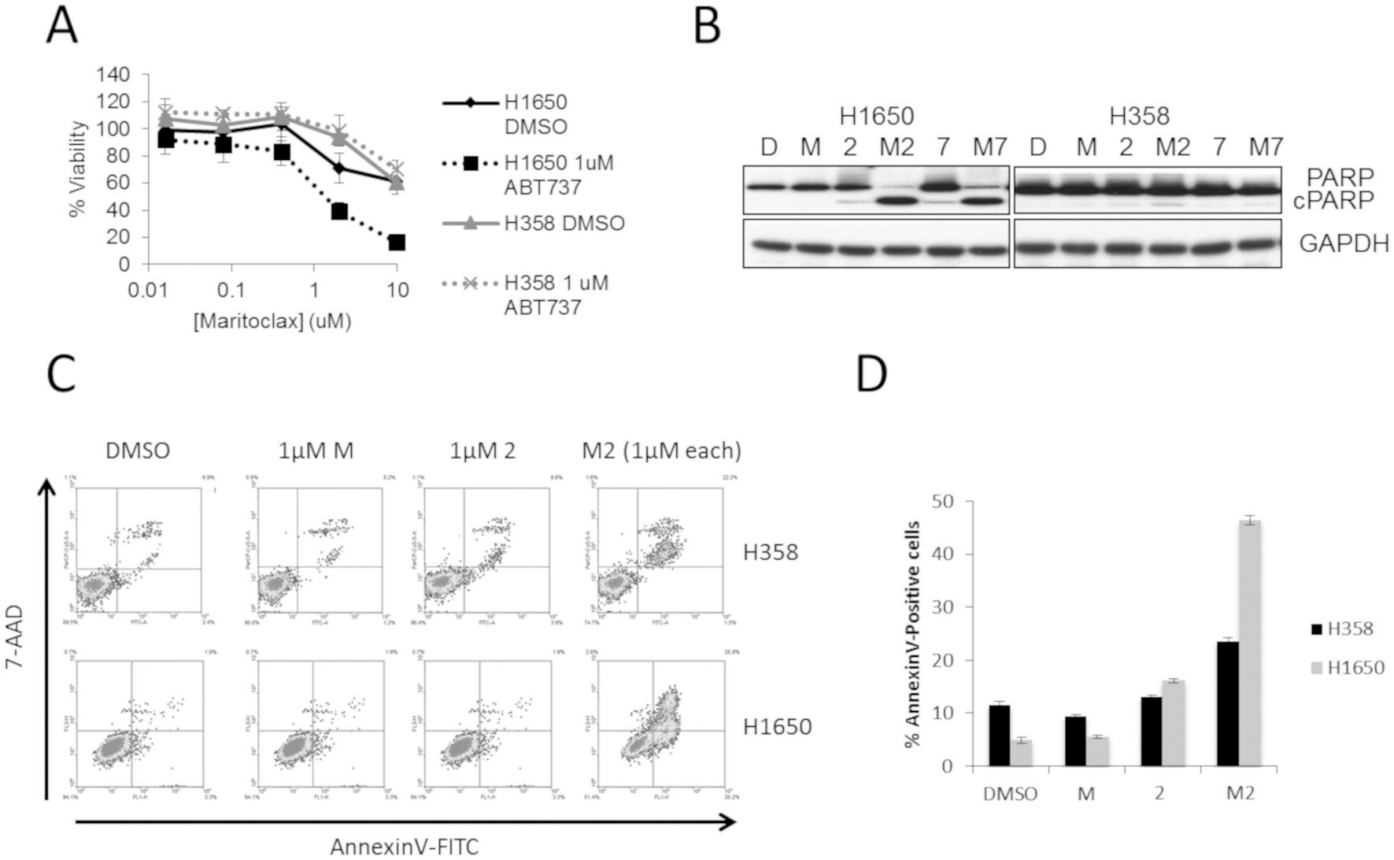
Maritoclax sensitizes H1650 cells to apoptosis when combined with a BCL-2/xL inhibitor. (A) Synergistic (H1650) and non-synergistic (H358) cell lines were treated with varying maritoclax doses in the presence or absence of 1 μM ABT-737. Viability was measured by MTS after 48 hours of treatment (n = 3). (B) H1650 and H358 cell lines were treated alone or in combination with 1 μM maritoclax (M) and 1 μM ABT-263 (2) or 1 μM ABT-737 (7) for 24 hours. PARP cleavage was evaluated by western blot. (C-D) H1650 and H358 cells were treated with 1 μM maritoclax (M) and 1 μM ABT-263 (2) alone and in combination (M2). After 24 hours, apoptosis was measured using an Annexin-V-FITC/7-AAD assay.

Anti-apoptotic BCL-2 proteins besides MCL-1 (i.e. BCL-xL) can compensate for the loss of MCL-1 and prevent apoptosis [18]. Therefore we hypothesized that the efficacy of maritoclax could be enhanced in these cells by combining it with a BCL-2/BCL-xL inhibitor. We evaluated the effect of combining maritoclax and the BCL-2/BCL-xL inhibitor, ABT-263 (navitoclax) in our panel of NSCLC cell lines. We utilized the Bliss sum method to estimate synergy and compare cell lines [26]. The panel of NSCLC cell lines used above was treated in a 5x5 matrix of maritoclax and ABT-263 alone or in combination for 48 hours followed by cell viability measurements (Fig 3A and 3B). H358 cells had the lowest bliss score (Fig 3), which suggested no synergy between the two drugs was occurring in these cells (Fig 3A). While H1650 and H1975 produced Bliss scores ~2-fold higher than other cell lines (Fig 3C), suggesting that the drug combination produced a sizable synergistic effect in these cells. To verify this result, we selected the most synergistic and least synergistic cell lines from the panel, H1650 and H358, respectively. These cells were treated with varying concentrations of maritoclax in the presence or absence of ABT-737, a BCL-2/xL inhibitor and a parent compound to ABT-263. Like ABT-263, a synergistic effect was observed between maritoclax and ABT-737 (Fig 4A).

We next sought to determine if the combination enhanced apoptosis in these cells over single-agent treatments alone using the cell lines with the highest and lowest Bliss sum score, H1650 and H358, respectively. The combination of maritoclax and ABT-263 or ABT-737 induced high levels of PARP cleavage after 24 hours on H1650 cells, while there was no effect on PARP cleavage in H358 cells (Fig 4B). The combination of maritoclax and ABT-263 induced apoptosis in approximately 50% of H1650 (Fig 4C and 4D) and H1975 (S3 Fig) cells (~10 and 5 fold increases compared to the DMSO control treatment, respectively). As a comparison, only 20% of H358 cells (~2 fold compared to the DMSO control treatment) were undergoing apoptosis after 24 hours, as determined by Annexin-V staining (Fig 4C and 4D). Similar changes in Caspase 3/7 activity were observed for the three cell lines following the combination treatment (S3C Fig).

#### AKT phosphorylation and EGFR mutation status are markers for BCL-2 and MCL-1 inhibitor combination treatment

**Fig 5:**
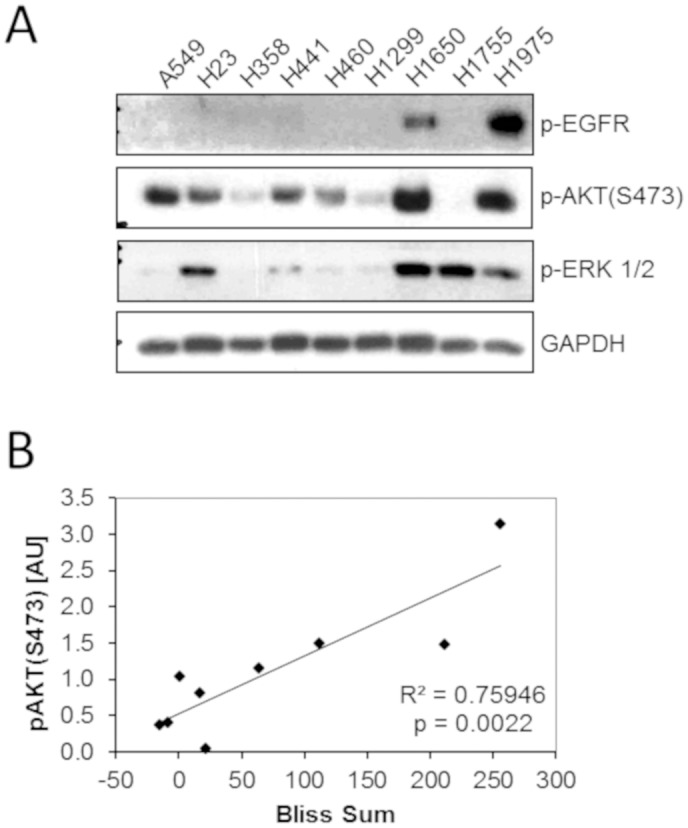
The extent of AKT signaling correlates with NSCLC sensitivity to the combination of maritoclax and ABT-263. (A) Expression of phosphorylated EGFR, AKT, and ERK1/2 kinases in NSCLC cells lines were evaluated by western blot. (B) The phosphorylated AKT signal from the western blot was quantified by densitometry and compared to the Bliss Sum value for each cell line as determined in Fig 2.

Because the two most sensitive cell lines (H1650 and H1975) to the maritoclax and ABT-263/737 combination were EGFR-mutated and erlotinib-resistant, we wanted to evaluate if EGFR-specific signaling was involved in sensitizing these cells to this combination. Endogenous levels of phosphorylated EGFR (Y1068), AKT (S473), and ERK (T202, Y204) were assessed by western blot in all nine cell lines. As expected, H1650 and H1975 cells had high levels of endogenous phosphorylated EGFR and AKT (Fig 5A). We observed a strong positive correlation (R^2^ = 0.759) between phosphorylated AKT and synergy (Bliss sum value) to the maritoclax/ABT263 combination (p = 0.0022) in the panel of cell lines (Fig 5B). This suggests that a high level of phosphorylated AKT is a predictive indicator of tumor cell sensitivity to the combination of maritoclax and a BCL-2/xL inhibitor.

## Discussion

We set out to study the efficacy of the MCL-1 inhibitor maritoclax in NSCLC. Based on previous reports, we characterized maritoclax alone and in combination with the BCL-2/xL-specific inhibitor navitoclax in a panel of nine NSCLC cell lines with mutation profiles proportional to that seen in lung cancer patients. We report three important findings, 1) sensitivity of NSCLC cell lines to MCL-1 inhibition was independent of MCL-1 expression and driver mutation status in these cells, 2) the combined inhibition of MCL-1 and BCL-2/xL with maritoclax and navitoclax (ABT263) is superior to either single drug treatment, especially in erlotinib-resistant EGFR mutated NSCLC cells, and 3) phosphorylated AKT is a marker for sensitivity to the maritoclax and navitoclax combination therapy.

It has been shown that targeting MCL-1 by siRNA knock-down or small molecule inhibition can effectively kill solid tumor cells [18,22]. These studies provide evidence that MCL-1 inhibitors can be effectively applied to solid tumors, but the data is limited and only in those known to be dependent on MCL-1 for survival. Because lung cancer is a genetically heterogeneous disease, it is essential to understand how these drugs perform in various different backgrounds. We found that single-agent maritoclax was effective at increasing MCL-1 degradation and inhibiting growth in cell lines with diverse oncogenic driver genes. IC_50_ values for eight of the nine cell lines were between ~1–4 μM, which is in the range of IC_50_ values for maritoclax-sensitive cells in other types of cancer [21, 22].

We and others [18] have demonstrated that many NSCLC cell lines express multiple anti-apoptotic BCL-2 family proteins (i.e. MCL-1, BCL-2, and BCL-xL). We hypothesized that a BCL-2/BCL-xL inhibitor (navitoclax) paired maritoclax would synergistically reduce cell growth and viability. This combination was most potent in EGFR-mutated, erlotinib-resistant NSCLC cell lines. It should be noted that our panel of cell lines only included erlotinib-resistant NSCLC cell lines (H1650 and H1975) and no cell lines with erlotinib sensitive EGFR mutations were tested.

EGFR and its ligand EGF contribute to drug resistance and are associated with activation of MCL-1 expression. Erlotinib-resistant lung cancer cells show enhanced sensitivity to navitoclax when combined with an EGFR inhibitor [27,28]. Additionally, neuroblastoma cells can acquire resistance to the BCL-2 inhibitor ABT-737 by upregulating EGFR and develop a dependence on MCL-1, which can be effectively countered by combining erlotinib with a BCL-2 inhibitor [29]. EGFR knockdown in these resistant cells disrupts the BIM-MCL-1 interaction and re-sensitizes these cells to ABT-737 [29]. Exogenous EGFR-targeted treatment activates MCL-1 expression and protects against apoptosis in breast and NSCLC cancer cells [30–32]. Together these findings suggest that the EGFR is essential for maintaining growth and preventing apoptosis in cancer cells, and up-regulation of MCL-1 is an important mechanism through which EGFR functions. Based on the context of our results, targeting MCL-1 with maritoclax in partnership with navitoclax could be a direct and efficient approach to treat patients with acquired resistance to first-generation EGFR inhibitors.

Aside from EGFR mutations, we also identified AKT signaling as a marker for sensitivity to the maritoclax and navitoclax combination therapy. Activated AKT can phosphorylate BAD and culminates in a pro-survival state [33]. Therefore, if survival is dependent on AKT and BAD, perturbation of other anti-apoptotic BCL-2 members could alter the state of those cells and push them towards death. In fact, the PIK3 inhibitor GDC-0941 has been shown to inhibit AKT phosphorylation of BAD, decrease MCL-1 expression, and inhibit the growth of glioblastoma cells in synergy with ABT-263 [34]. Based on our data, it seems reasonable that a similar effect could be occurring in the lung cancer cell.

AKT signaling has been implicated in ABT-263 resistance. Recently it was reported that ABT-263 treatment alone can enhance the MCL-1 mRNA and protein levels in hepatocellular carcinoma, and these cells could be sensitized to ABT-263 by inhibition of the AKT pathway [35]. These data suggested that the AKT pathway acted to promote resistance to ABT-263 mainly through stabilization of MCL-1 expression. Here we show that directly targeting MCL-1 can effectively sensitize NSCLC cells with high AKT expression, to ABT-263. This observation could be important for selecting those patients who are most likely to have a clinically favorable response to the combined inhibition of MCL-1 and BCL-2/xL.

In conclusion, we extensively characterized the MCL-1 inhibitor, maritoclax, in NSCLC cell lines. We found that the drug was broadly effective *in vitro*, and it functioned in an MCL-1-dependent manner. The efficacy of the drug can be enhanced by pairing it with the BCL-2/xL inhibitor ABT-263. This combination was most effective in cells with high levels of activated AKT.

## Supporting information

S1 Fig Maritoclax inhibits MCL-1 expression in many lung cancer cell lines. A concentration-dependent inhibition of MCL-1 was observed in four non-small cell lung cancer cells lines. (A-B) Indicated cells were treated with varying concentrations of maritoclax or vehicle control (V) for 24 hours. Proteins were harvested and probed for MCL-1 protein expression. (C) H1299 cells were treated with 1 μM maritoclax or DMSO control for the indicated times, prior to protein harvest and western blot analysis for MCL-1.
(TIF)

S2 Fig Maritoclax induces Caspase 3/7 activity in H23 cells. H23 cells were treated with the indicated concentrations of maritoclax for 24 hours, prior to measurement of Caspase 3/7 activity. (* = p<0.05, ** = p<0.01, *** = p<0.001).
(TIF)

S3 Fig Combined maritoclax and BCL-2/xL inhibition induce apoptosis in the NSCLC cell lines H358 and H1975. (A-B) The indicated cell lines were treated with maritoclax (1 μM) and ABT-263 (1 μM) alone or in combination for 24 hours. Apoptotic (Annexin-V positive) cells were measured using flow cytometry. (C) Each cell line was treated with the same concentration of drugs as in (A-B) for 24 hours, prior to measurement of Caspase 3/7 activity.
(TIF)
