# Supplementary figures and images for: EGFR mutations and AKT phosphorylation are markers for sensitivity to combined MCL-1 and BCL-2/xL inhibition in non-small cell lung cancer
Source: PLoS One. 2019 May 31;14(5):e0217657. doi: 10.1371/journal.pone.0217657 (PMC6544263; doi:10.1371/journal.pone.0217657)

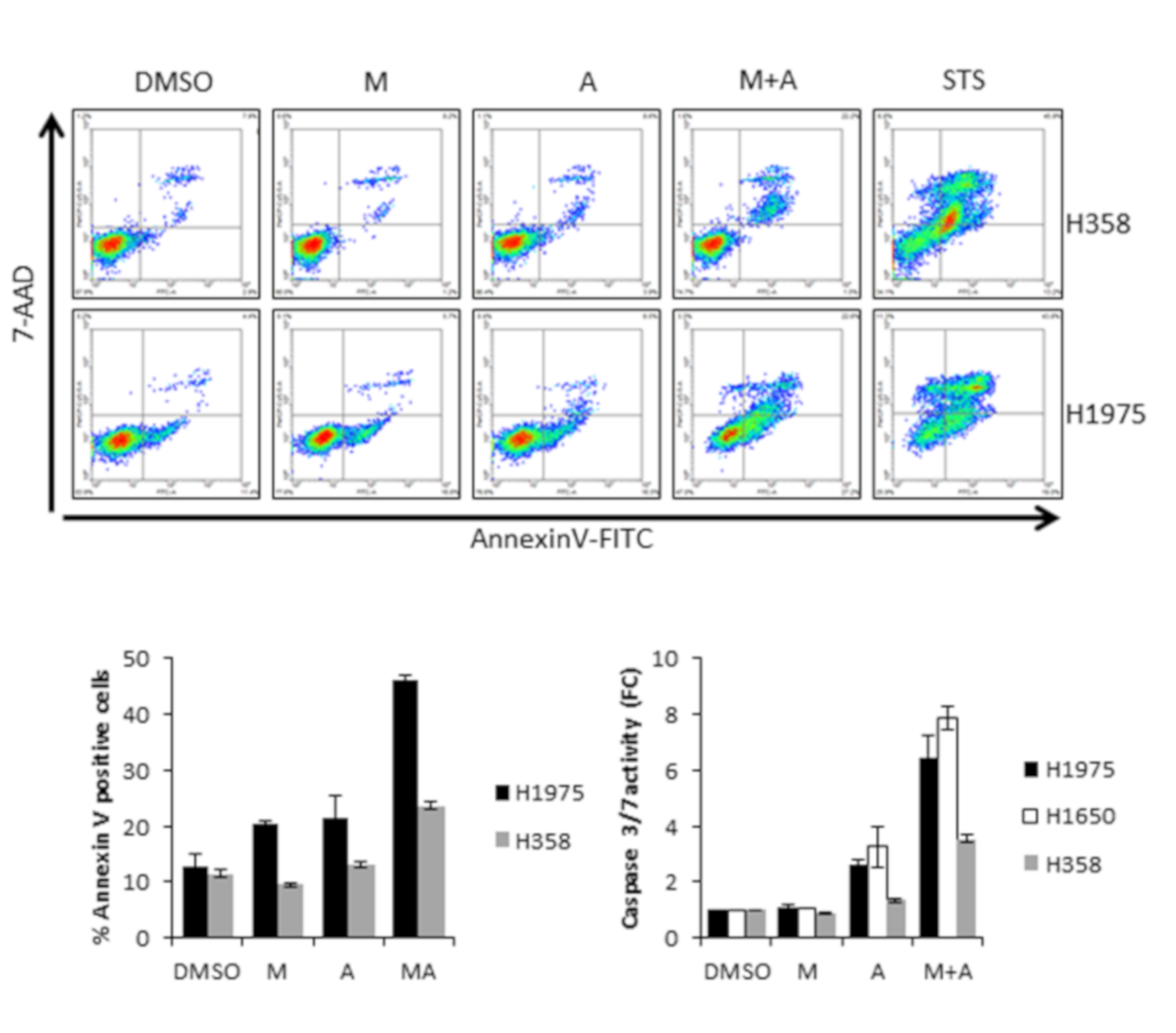

Supplement: S3 Fig — (A-B) The indicated cell lines were treated with maritoclax (1 μM) and ABT-263 (1 μM) alone or in combination for 24 hours. Apoptotic (Annexin-V positive) cells were measured using flow cytometry. (C) Each cell line was treated with the same concentration of drugs as in (A-B) for 24 hours, prior to measurement of Caspase 3/7 activity. (TIF) [file pone.0217657.s003.tif]
